# Mixed Ovarian Carcinoma Comprising Clear Cell Carcinoma and Endometrioid Carcinoma: A Case Report

**DOI:** 10.7759/cureus.73483

**Published:** 2024-11-11

**Authors:** Jason D Susanto, Praisela S Hendrieta Nelwan, Fajar L Gultom, Batara I Sirait, Iwan K Effendi

**Affiliations:** 1 General Medicine, Faculty of Medicine, Universitas Kristen Indonesia, Jakarta, IDN; 2 Obstetrics and Gynecology, Universitas Kristen Indonesia General Hospital, Jakarta, IDN; 3 Anatomical Pathology, Universitas Kristen Indonesia, Jakarta, IDN; 4 Anatomical Pathology, Mochtar Riady Comprehensive Cancer Center (MRCCC) Siloam Hospital Semanggi, Jakarta, IDN; 5 Obstetrics and Gynecology, Universitas Kristen Indonesia, Jakarta, IDN; 6 Obstetrics and Gynecology, Mochtar Riady Comprehensive Cancer Center (MRCCC) Siloam Hospital Semanggi, Jakarta, IDN; 7 Obstetrics and Gynecology, Universitas Trisakti, Jakarta, IDN

**Keywords:** epithelial ovarian cancer, mixed ovarian carcinoma, ovarian cancers, ovarian clear cell carcinoma, ovarian endometrioid adenocarcinoma

## Abstract

We present a unique case of mixed ovarian carcinoma, including both clear cell carcinoma (CCC) and grade 1 endometrioid carcinoma (EC) in a 49-year-old woman. The patient had no complaints of pain but presented with an enlarged abdomen, necessitating a thorough diagnostic assessment. Histopathological evaluation revealed an unusual histological juxtaposition of CCC coexisting with grade 1 EC found in an ovarian mass, resulting in diagnostic and therapeutic challenges. A comprehensive approach was used to treat this particular case of mixed ovarian cancer, which included surgical procedures and adjuvant treatments. This case emphasizes the significance of identifying and assessing mixed ovarian carcinomas in order to develop customized treatment plans, as well as the necessity of further research into the basic underlying causes of such unusual presentations.

## Introduction

Ovarian cancer, categorized as the second-most common malignancy of the female reproductive system in the United States, is divided into nonepithelial ovarian cell cancers and epithelial ovarian cell cancers, representing 10% and 90% of cases, respectively [1]. These cancers are mostly found later in life and discovered late because of their nonspecific clinical presentation, with poor prognosis. The most common histological findings of epithelial ovarian carcinoma (EOC) are papillary serous carcinoma (70% of cases), followed by endometrioid carcinoma (EC) (20%-25% of cases) and clear cell carcinoma (CCC) (5%-10% of cases) [2]. EOC is considered the most lethal gynecologic malignancy [3].

Mixed endometrial carcinomas comprise two or more distinct histotypes of EC, with the most frequent findings being a combination of endometrioid and serous carcinomas, followed by a combination of EC and CCC [4]. Endometriosis is considered a benign gynecologic disease distinguished by the presence of stroma and endometrial glands outside of the uterus and is a known risk factor for EOC. One study described a relationship between ovarian cancer and endometriosis in which either a transition occurs, where endometriotic lesions turn into an invasive ovarian carcinoma, or there is a co-existence of endometriosis and ovarian carcinoma without any transition [2].

Clear cell ovarian carcinoma (CCOC), especially in the late stage, is eminently insensitive to gold-standard chemotherapeutic agents, resulting in a worse prognosis than that of more frequent serous adenocarcinoma or EC [3]. There are still many unknown mechanisms of mixed endometrial carcinomas, especially mixed EC and CCC, owing to the rarity of their detection. By presenting this case, we aim to provide an example of a rare type of mixed ovarian carcinoma comprising mixed EC and CCC, with a special emphasis on the case’s histopathology profile, which may provide guidance for treating such cases.

## Case presentation

A 49-year-old woman presented with an enlarged abdomen since the last three months prior as her chief complaint, which she said was growing bigger every day without pain. The patient denied any other intestinal problems. The patient had been married for 17 years and did not have children. She had a history of a right ovarian cyst, which was extracted approximately one year prior, and was currently taking 5 mg of amlodipine daily to treat hypertension.

On physical examination, the patient had a body weight of 76 kg and a height of 155 cm. The patient was alert, oriented, and looked well without any feelings of pain but felt discomfort because of her enlarged abdomen. Her vital signs were as follows: a blood pressure of 138/94 mmHg, a heart rate of 117 beats per minute, a respiratory rate of 22 breaths per minute, and a temperature of 36.7^°^C. Her abdomen appeared enlarged and without any tenderness from palpation and percussion. Edema was noted on the left lower extremity.

The patient’s complete blood count indicated normocytic normochromic anemia and slight thrombocytosis. The patient also had a high erythrocyte sedimentation rate and CA-125 count (Table 1). 

**Table 1:** Laboratory test results CA-125: Cancer antigen-125

Laboratory test	Value	Reference value
Hemoglobin (g/dL)	10.3	12.0 – 16.0
Hematocrit (%)	30.7	37.0 – 47.0
Leucocyte (10^3^/µL)	9.6	4.0 – 10.0
Thrombocyte (10^3^/µL)	492	150 – 400
Erythrocyte (10^6^/µL)	3.81	4.20 – 5.40
Erythrocyte sedimentation rate (mm/hour)	110	≤ 20
Mean corpuscular volume (fL)	80.6	81.0 – 96.0
Mean corpuscular hemoglobin (pg)	27.0	27.0 – 36.0
Mean corpuscular hemoglobin concentration (g/L)	33.6	31.0 – 37.0
D-dimer (ng/mL)	203	≤ 250
CA-125 (U/mL)	1228	0 – 35

A computed tomography (CT) scan of the abdomen and pelvis with IV contrast showed a complex bilateral adnexal cystic mass, with the right mass measuring 12x13x13 cm and the left mass measuring 68x62x72 mm, which pressurized the surrounding organs. Also shown was a simple cortical renal cyst measuring 8 mm.

The patient was suggested to have an operation for debulking and frozen section, and if the results showed malignancy, continuation with midline incision. The patient agreed for the operation. During the operation, the frozen section was done, and the result showed a figure tending towards endometrioid carcinoma from the right ovary and an endometriosis cyst from the left ovary. The operation was continued with surgical staging, dissection of bilateral pelvic and aortic lymph nodes, omentectomy, and appendectomy. The operation was successful, and the patient was then sent home six days post-operatively in good condition and was told to return for further treatment after the histopathology report was done or at any time if necessary.

**Figure 1:**
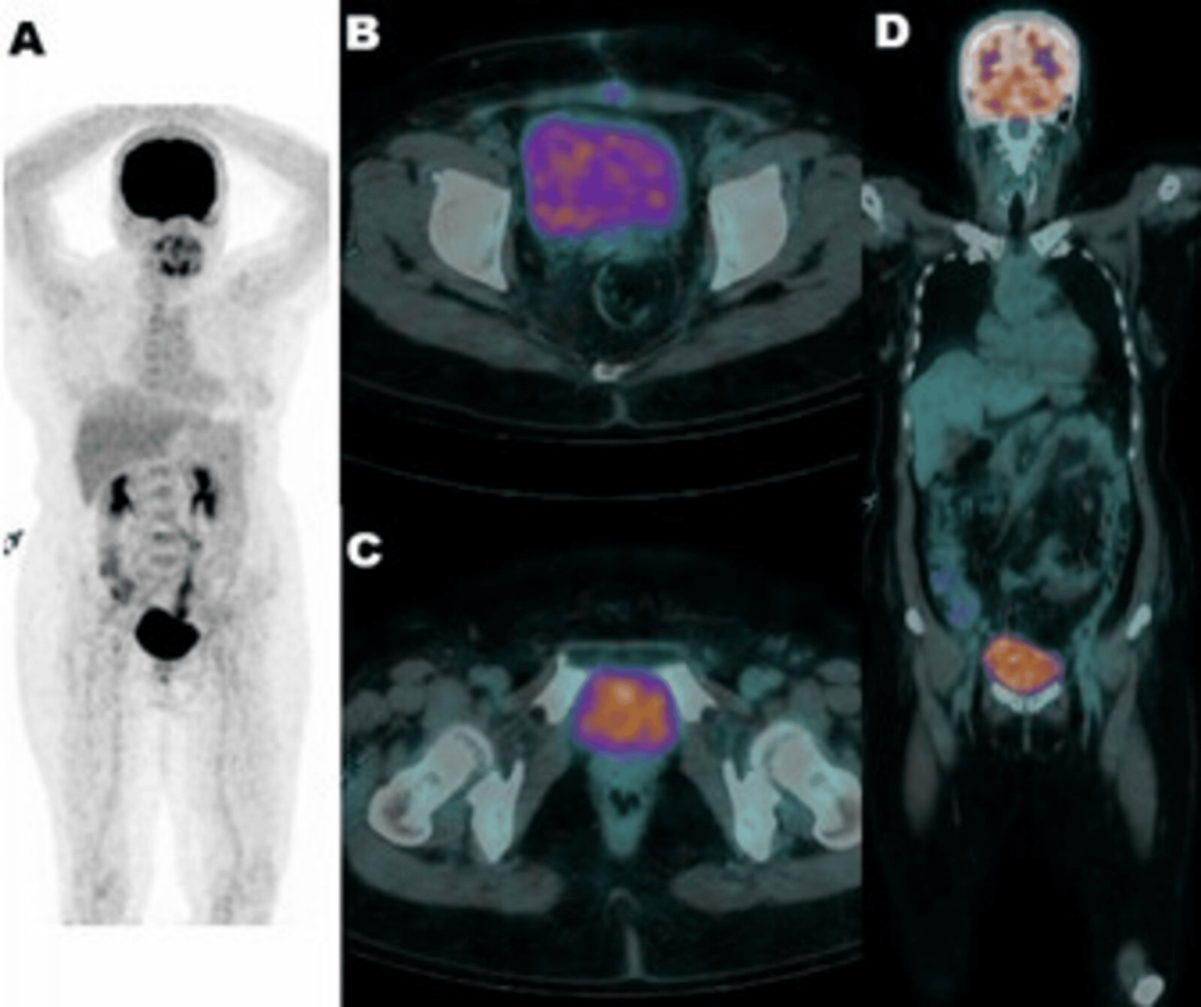
Baseline [18F] fluorodeoxyglucose positron emission tomography/computed tomography (PET/CT) performed for the detection of metastasis (A) maximum intensity projection, (B) pelvic transaxial PET/CT, (C) rectal transaxial PET/CT, and (D) head-abdomen coronal PET/CT.

**Figure 2:**
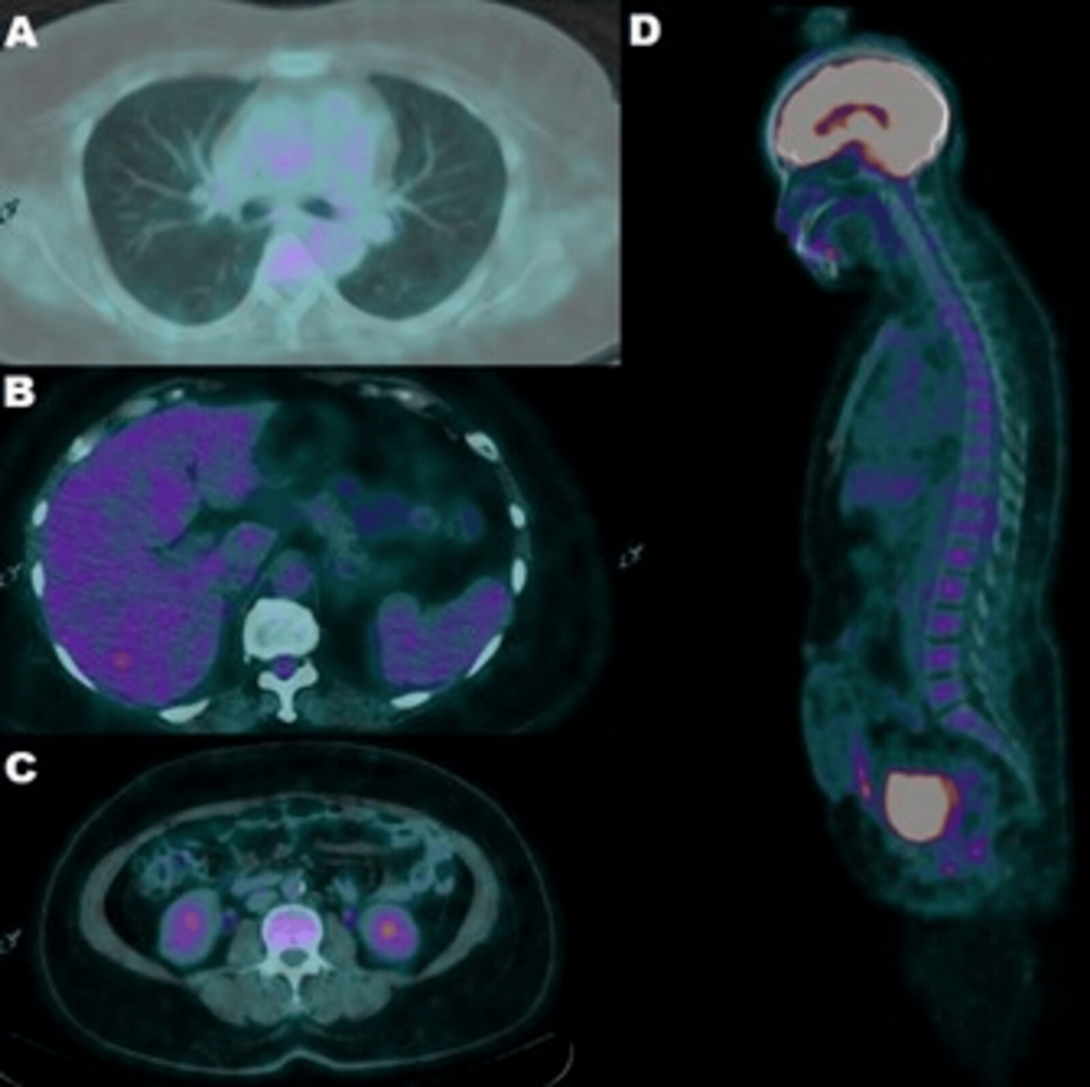
Continued baseline [18F] fluorodeoxyglucose positron emission tomography/computed tomography (PET/CT) performed for the detection of metastasis (A) thoracic transaxial PET/CT, (B) hepatic transaxial PET/CT, (C) renal transaxial PET/CT, and (D) head-abdomen sagittal PET/CT.

Before being sent home, the patient had a positron emission tomography-computed tomography (PET/CT) scan with 18F fluorodeoxyglucose (FDG) that showed the uterus bed and vagina without apparent suspicion of malignancy residue, with clear findings from the peritoneum-mesenterium. In addition, there was no apparent suspicion of metastasis from the abdomen-pelvis-inguinal lymph nodes, both lungs, liver, or bones, as seen in Figures 1, 2.

**Figure 3:**
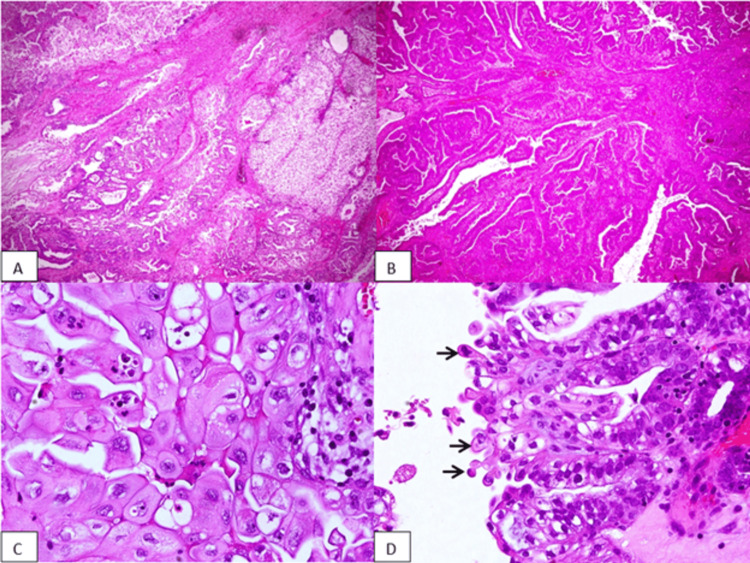
Hematoxylin and eosin (HE) stained section of the right ovary (A) tumor cells arranged in solid, tubulocystic, and papillary patterns (HE, 40X), (B) tumor cells arranged in back-to-back endometrioid glands (HE, 40X), (C) tumor cells with squamous differentiation (HE, 400X), and (D) hobnail cells in clear cell component (arrows) (HE, 400X).

**Figure 4:**
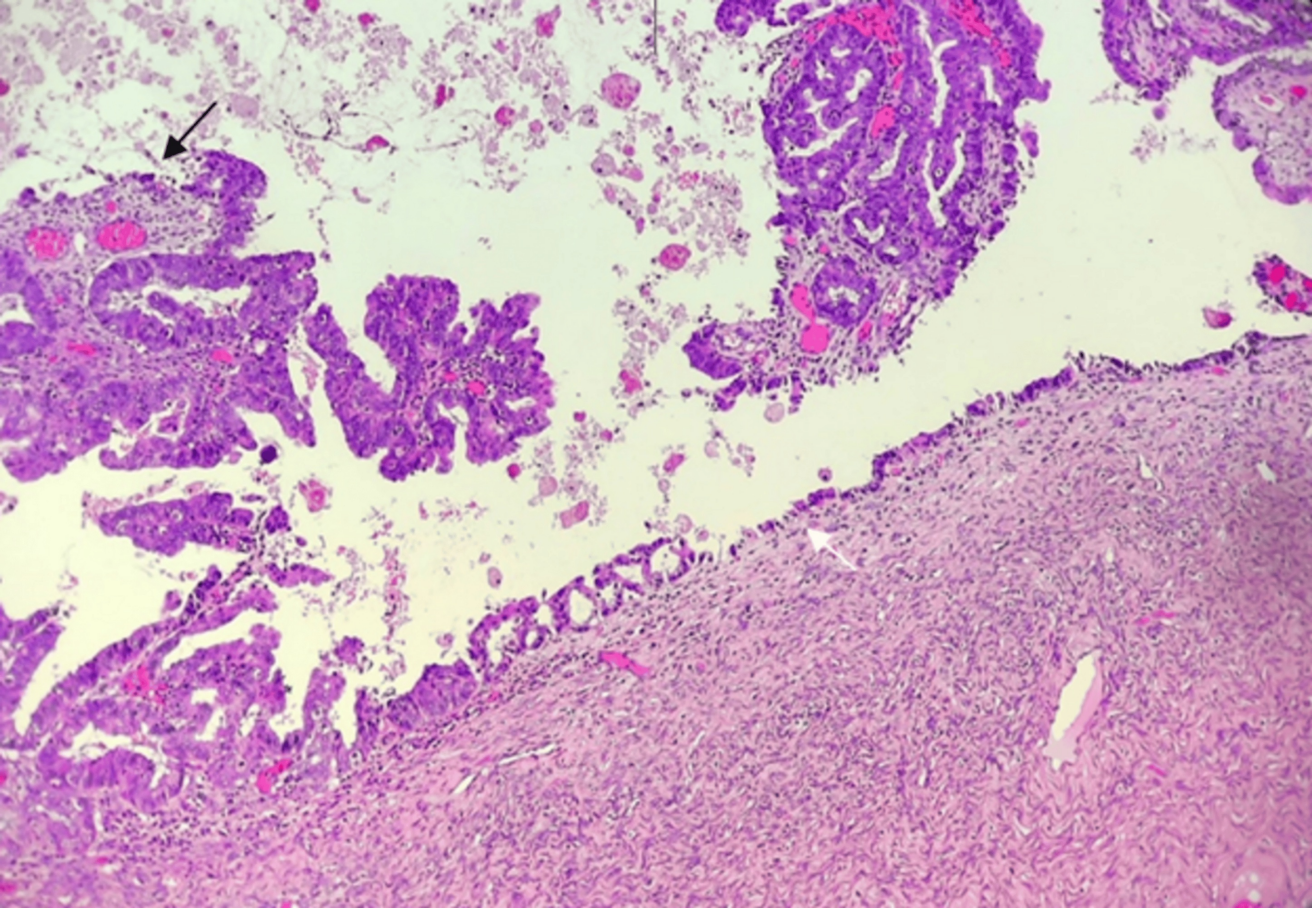
Hematoxylin and eosin stained section (100X) showing grade 1 endometrioid cancer (black arrow) with endometriosis cyst (white arrow) from the left ovary

The histopathology report of the right ovary showed mixed carcinoma comprising CCC with grade 1 EC, without any invasion to the lymphovascular and tubal tissue without significant abnormalities, as seen in Figure 3. The left ovary showed grade 1 endometrioid cancer, without any invasion to the lymphovascular, and tubal tissue without significant abnormalities but the appearance of an endometriosis cyst in the background, as seen in Figure 4.

The patient was then planned for adjuvant chemotherapy treatment, which she is still receiving currently. The patient is expected to have a good outcome after completing the planned treatment.

## Discussion

Clear cell ovarian carcinoma (CCOC) is an exclusive pathological type of EOC and an aberrant type of ovarian cancer [5]. CCOC is also categorized as the second-most frequent subtype of EOC, accounting for 1%-12% of EOC cases, depending on ethnicity; the prevalence of CCOC in Asian, Black, and White female populations is 11.1%, 3.1%, and 4.8%, respectively [3,6]. CCC is distinguished by the presence of clear or often eosinophilic cytoplasm and hobnail cells, which exhibit different histologic patterns (e.g., solid, papillary, or tubulocystic), with all of these characteristics identifiable within a single neoplasm. These histologic features are similar to those of CCC, which develops in the ovary, uterus, cervix, or vagina [7,8]. Although there is no definitive molecular finding that is completely sensitive to CCC, immunohistochemistry may find somatic mutations in the ARID1A, TP53, PIK3CA, PIK3R1, PPP2R1A, and KRAS genes [7].

Endometriosis is known as a benign and chronic gynecologic disease that is distinguished by the growth of stroma and endometrial glands outside of the uterus. Endometriosis is also known to potentially develop into EOC, with a rate of malignancy of 0.3%-0.8% [2]. Two distinct relationships between ovarian cancer and endometriosis have been established. The first is a transition of endometriosis lesions into an invasive ovarian carcinoma, and the second is a coexistence between endometriosis and ovarian cancer without any transition. Epithelial ovarian cancers that are correlated with endometriosis include EC and CCC [2]. Ovarian cancers are known as a type of cancer in postmenopausal women; they are rare in women under 40 years of age but are commonly found in older women between the ages of 60-65 years in developed countries [9].

Although CCOC is rare, its prevalence is high in the Japanese population, which may be associated with differences in genetic factors and social-environmental factors, including this population’s shifting of age at menarche and menopause (i.e., earlier age of menarche and later age of menopause). In addition, changes in diet, marital status, smoking status, use of oral contraceptives, and a remarkably decreased pregnancy rate may also lead to an increased risk of CCOC. These factors augment the number of ovulations and, thus, menstruations in a woman’s lifetime, and those with increased rates are considerably more susceptible to developing endometriosis, which is notorious for leading to EC and CCC [10]. The risk of malignant ovarian cancer is also increased in women with solid masses or complex cysts, with the 3-year risk of cancer reaching 430 per 1000 women based on the patient’s age and ultrasonography findings [11]. A meta-analysis by Jiang et al. showed that infertility is also a significant risk factor for ovarian cancer, with a 51% risk increase [12]. In our case, the patient was a 49-year-old woman with nulliparity despite being married for 17 years without using any contraceptives. She also had a history of a right ovarian cyst approximately one year prior that had been extracted. Histopathological findings showed the appearance of an endometriosis cyst on the left ovary. Many of her characteristics matched the risk factors of ovarian cancer, including age, nulliparity, history of extracted ovarian cyst, current endometriosis cyst on the left ovary, and the possibility of infertility.

Histopathological findings of CCC in the ovary generally comprise a mixture of solid, papillary, and tubulocystic architectural patterns with hobnail or cuboidal cells and eosinophilic cytoplasm at the lining epithelial cells, low mitotic activity, and infrequent high nuclear grade. However, in reality, most cases are found with histopathological findings of other types of ovarian and endometrial carcinomas, which may present clear cell changes, possibly leading to diagnostic indecision. Therefore, immunohistochemistry is used as a diagnostic tool for CCC, where Napsin-A is considered an accurate and trustworthy marker to identify and distinguish CCC from non-CCC in the endometrium and ovary. In a study by Nili et al., the precision, specificity, and sensitivity of Napsin-A in diagnosing endometrial and ovarian CCC were 83%, 100%, and 83%, respectively, whereas its sensitivity for ovarian CCC and endometrial CCC was 85% and 80%, respectively [13,14]. Napsin-A is an enzyme of the pepsin family called cytoplasmic aspartic protease, which is predominantly expressed in the lung, kidney, pancreas, and also some types of epithelial neoplasms, including the gynecologic CCC [15].

EC resembles endometrioid adenocarcinoma of the endometrium, which accounts for 10% of primary ovarian carcinomas that develop in women around 55 years of age, especially women with endometriosis or endometriotic cysts. Histopathological findings of EC in the ovary include confluent (back-to-back) glands morphologic pattern, stromal invasion, cytoplasmic mucin, intracytoplasmic vacuoles, squamous metaplasia, and oncocytic and clear cell changes, and the presence of adenofibroma or endometriosis may be found in the background [16,17]. Sexual steroid hormone receptors play a significant role in ovarian cancer, especially EC, which usually presents with a higher frequency of positive results for androgen receptor (AR), estrogen receptor alpha (ER), and progesterone receptor (PR). These receptors play a key role in the growth and treatment of endometrioid cancer through their function of regulating signaling pathways affiliated with cell proliferation, apoptosis, and transition of the epithelial-mesenchymal and invasiveness, which are necessary and crucial for tumor progression. Several studies have demonstrated the expression of PR as a plausible prognostic biomarker in EC. Therefore, PR staining is often used in immunohistochemistry as a diagnostic tool for EC [18,19].

**Figure 5:**
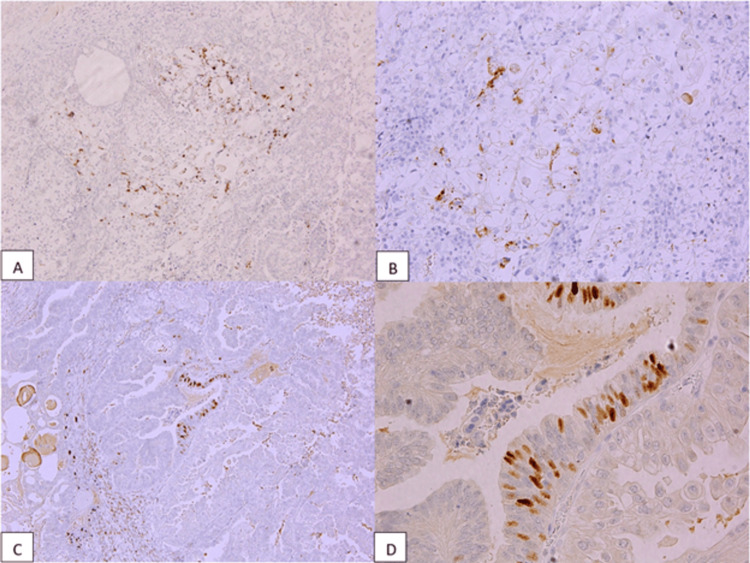
Immunohistochemistry (IHC) of the right ovary (A, B) immunostain for Napsin-A showing positive cystoplasmic in clear cell component (brown stained) (Napsin-A IHC 40X and 100X, respectively); (C, D) immunostain for progesterone receptor (PR) showing positive nuclear in endometrioid component (brown stained) (PR IHC 100X and 400X, respectively).

In our case, the patient’s histopathology report showed mixed carcinoma comprising CCC with EC of the right ovary and EC with endometriosis cyst of the left ovary. Hematoxylin and eosin (H&E) staining was used, with histopathological findings showing solid, tubulocystic, and papillary structured tumor masses with hobnail cells on the epithelial lining, which characterize CCC and squamous differentiated endometrioid cancer from the right ovary. H&E staining was also done for the left ovary, which showed endometrioid cancer with an endometriosis cyst. Immunohistochemistry staining was done to verify that this case was a mixed ovarian carcinoma, with a positive result for Napsin-A indicating CCC and a positive result for PR indicating EC from the right ovary. Both positive results are shown as indicated by the appearance of brown staining in Figure 5.

Early-stage ovarian CCC usually has a great prognosis; however, late-stage ovarian CCC is well known to be less sensitive to gold-standard platinum-based chemotherapy, resulting in a worse prognosis than that of other more common carcinomas such as serous adenocarcinoma and endometrioid adenocarcinoma at the same stage [2,3]. Meanwhile, the prognosis of ovarian EC is mostly good. However, there are still many unsettled problems for CCC and EC as tumors, especially in diagnosis and treatment selection, as they are closely related to endometriosis. Therefore, these tumors require further research, as both have distinct histopathological characteristics and worse prognoses when comorbid with endometriosis [2]. However, several studies have reported that endometriosis does not affect the prognosis of either ovarian CCC or EC [20].

In our case, the patient’s PET/CT scan showed no apparent suspicion of malignancy residue or metastasis, and she was then planned for adjuvant chemotherapy treatment with the expectation of having a good outcome after completing the planned treatment.

## Conclusions

CCOC is a special and rare type of ovarian cancer with a poor prognosis in its late stage. The presence of endometriosis may contribute to the development of mixed ovarian cancer of CCC and EC. Early diagnosis and prompt treatment are essential for staging and prognosis, with histopathology as the gold standard for identification. Definitive diagnosis requires a thorough approach combining histopathological and immunohistochemistry with specialized staining techniques.

## References

[1] Torre LA, Trabert B, DeSantis CE (2018). Ovarian cancer statistics, 2018. CA Cancer J Clin.

[2] Zhou L, Yao L, Dai L (2021). Ovarian endometrioid carcinoma and clear cell carcinoma: A 21-year retrospective study. J Ovarian Res.

[3] Tang H, Liu Y, Wang X, Guan L, Chen W, Jiang H, Lu Y (2018). Clear cell carcinoma of the ovary: clinicopathologic features and outcomes in a Chinese cohort. Medicine (Baltimore).

[4] Köbel M, Meng B, Hoang LN (2016). Molecular analysis of mixed endometrial carcinomas shows clonality in most cases. Am J Surg Pathol.

[5] Ji JX, Cochrane DR, Negri GL (2022). The proteome of clear cell ovarian carcinoma. J Pathol.

[6] Angelina YA, Tjokroprawiro BA (2023). Advanced stage clear cell ovarian carcinoma mimicking uterine sarcoma without gross residual tumor during primary surgery: a case report. Clin Med Insights Case Rep.

[7] Hagemann IS, Deng W, Zaino RJ (2023). Mixed clear cell/endometrioid and clear cell/serous carcinoma of the uterus are clinicopathologically similar to pure clear cell carcinoma: An NRG Oncology/Gynecologic Oncology Group (GOG-210) study of 311 women. Gynecol Oncol.

[8] Abdulfatah E, Sakr S, Thomas S (2017). Clear cell carcinoma of the endometrium: evaluation of prognostic parameters in a multi-institutional cohort of 165 cases. Int J Gynecol Cancer.

[9] Ali AT, Al-Ani O, Al-Ani F (2023). Epidemiology and risk factors for ovarian cancer. Prz Menopauzalny.

[10] Machida H, Matsuo K, Yamagami W (2019). Trends and characteristics of epithelial ovarian cancer in Japan between 2002 and 2015: A JSGO-JSOG joint study. Gynecol Oncol.

[11] Smith-Bindman R, Poder L, Johnson E, Miglioretti DL (2019). Risk of malignant ovarian cancer based on ultrasonography findings in a large unselected population. JAMA Intern Med.

[12] Jiang YT, Gong TT, Zhang JY, Li XQ, Gao S, Zhao YH, Wu QJ (2020). Infertility and ovarian cancer risk: evidence from nine prospective cohort studies. Int J Cancer.

[13] Nili F, Tavakoli M, Izadi Mood N, Saffar H, Sarmadi S (2020). Napsin-A expression, a reliable immunohistochemical marker for diagnosis of ovarian and endometrial clear cell carcinomas. Iran J Pathol.

[14] Rekhi B, Deodhar KK, Menon S (2018). Napsin A and WT 1 are useful immunohistochemical markers for differentiating clear cell carcinoma ovary from high-grade serous carcinoma. APMIS.

[15] Zhu B, Rohan SM, Lin X (2015). Immunoexpression of napsin A in renal neoplasms. Diagn Pathol.

[16] (2024). Pathology outlines- endometrioid carcinoma. https://www.pathologyoutlines.com/topic/ovarytumorendometrioidcarcinoma.html.

[17] Peres LC, Cushing-Haugen KL, Köbel M (2019). Invasive epithelial ovarian cancer survival by histotype and disease stage. J Natl Cancer Inst.

[18] Gómora MJ, Morales-Vásquez F, Pedernera E (2018). Sexual steroid hormone receptors profiles of ovarian carcinoma in Mexican women. Endocr Connect.

[19] Köbel M, Rahimi K, Rambau PF (2016). An immunohistochemical algorithm for ovarian carcinoma typing. Int J Gynecol Pathol.

[20] Paik ES, Kim TJ, Choi CH, Kim BG, Bae DS, Lee JW (2018). Clinical outcomes of patients with clear cell and endometrioid ovarian cancer arising from endometriosis. J Gynecol Oncol.

